# A comparative study on image-based snake identification using machine learning

**DOI:** 10.1038/s41598-021-96031-1

**Published:** 2021-09-27

**Authors:** Mahdi Rajabizadeh, Mansoor Rezghi

**Affiliations:** Department of Computer Science, Tarbiat Modares University, Tehran, Iran

**Keywords:** Herpetology, Computer science

## Abstract

Automated snake image identification is important from different points of view, most importantly, snake bite management. Auto-identification of snake images might help the avoidance of venomous snakes and also providing better treatment for patients. In this study, for the first time, it’s been attempted to compare the accuracy of a series of state-of-the-art machine learning methods, ranging from the holistic to neural network algorithms. The study is performed on six snake species in Lar National Park, Tehran Province, Iran. In this research, the holistic methods [k-nearest neighbors (*k*NN), support vector machine (SVM) and logistic regression (LR)] are used in combination with a dimension reduction approach [principle component analysis (PCA) and linear discriminant analysis (LDA)] as the feature extractor. In holistic methods (*k*NN, SVM, LR), the classifier in combination with PCA does not yield an accuracy of more than 50%, But the use of LDA to extract the important features significantly improves the performance of the classifier. A combination of LDA and SVM (kernel = 'rbf') is achieved to a test accuracy of 84%. Compared to holistic methods, convolutional neural networks show similar to better performance, and accuracy reaches 93.16% using MobileNetV2. Visualizing intermediate activation layers in VGG model reveals that just in deep activation layers, the color pattern and the shape of the snake contribute to the discrimination of snake species. This study presents MobileNetV2 as a powerful deep convolutional neural network algorithm for snake image classification that could be used even on mobile devices. This finding pave the road for generating mobile applications for snake image identification.

## Introduction

With around 81.410 to 137.880 deaths per year (https://www.who.int), snakes are among the top three dangerous animals for human. Out of 3848 known species of snakes, around 800 species are venomous, among which only about 50 species are fatal to human (https://www.reptile-database.reptarium.cz).

Identification of snakes is not easy; For example, those characters discriminating the non-venomous snake from the viperids (oval shaped head, round pupil, absence of a pit) occur in the elapid snakes either; while both viperids and elapids are venomous or fatal to the human. Hence, proper snake identification would entail herpetological skills that use body and head morphological features (color, pattern, shape, scalation, and etc.)^1^. Automated snake image identification is important from different points of view, most importantly, snake bite management. Auto-identification of snake images might help people avoid venomous snakes; besides, it can help healthcare providers plan a better treatment for patients bitten by snakes (see^2^).

Computer vision technology has developed rapidly in the field of automated image recognition and image classification^3^. Computer scientists apply different machine learning approaches for image classification^4^. Image classification using machine learning, consists of two phases: feature extraction and classification. In image classification the classes are predetermined; in summary, the process includes a training phase using the training data, and classification of the test data based on the trained model. By training, the predefined classes can be conceived of an available dataset that take the characteristic features of each image classes and shape a special description for each specific class^5^.

Application of machine learning (hereafter ML) for the identification of plants and animals’ images is growing rapidly (for a review see^6^). Recently, efforts have been made for image-based snake classification using ML^7–10^. In these researches a range of machine learning methods we used, from traditional ML classifiers, including k-nearest neighbors (hereafter *k*NN) and support vector machine (hereafter SVM), to the state of the art neural network algorithms like convolutional neural network (hereafter CNN). None of the former studies compared the accuracy of the traditional methods and neural networks in the classification of the snake images; nevertheless, there are challenges in the application of ML algorithms for the classification of snakes.

- First, because of the elongated and flexible body, snakes usually represent wide variations in the pose and deformation of the body. For example, in a limited image dataset of a snake, head or tail might be hidden under the body; besides, the body itself might be twisted in different directions and hence, the dorsal color pattern might show plenty of different ornamentations. So, acquiring features from the dorsal body pattern of snakes is quite challenging.
- Second, training a deep convolutional neural network requires a large image dataset. Unfortunately, not many specialized datasets are available for snakes. Regarding rare snakes this situation is even worse; On the other hand, since the museum specimens do not have natural color and pose, they are not applicable for incorporation in the whole body image datasets.

In this study, for the first time, it’s been planned to compare the accuracy of a series of state-of-the-art machine learning methods, ranging from the traditional to neural network algorithms. An attempt is made to evaluate the performance of these models in the classification of a limited, accessible series of snake images. For this purpose, the following guidelines are pursued:

- Minimum possible dataset: collecting snake images is not an easy task, and not all the images are necessarily taxonomically informative (*e.g.* art works). So, a dataset of 594 images of the whole body of six snake species were collected. Only those images in which at least 50% of the snake body was visible in the image were involved in the dataset.
- Feature extraction: to overcome the challenge of wide variations in the body pose of snakes in the images, a feature extraction method has been used in combination with traditional classifiers. Feature extraction is the process of representing a raw image in its reduced form to facilitate decision-making as to pattern classification^11^.
- Transfer learning: the size of our dataset is not optimum to train a state-of-the-art deep neural network model; To solve this issue, a transfer learning is used. In this method, off-the-shelf features extracted from a pre-trained network is transferred to a new CNN model^12^ for classification of snakes.
- Visualization of CNN hidden layers: to understand the learning process of a CNN model, a visualization method has been used, which visualizes the location of the discriminative regions of snakes’ images at each hidden layer^13^. Using this method, we can uncover snake identification process through a CNN model and also compare it with human experts.

## Results

### Principle component analysis (PCA)

**Figure 1:**
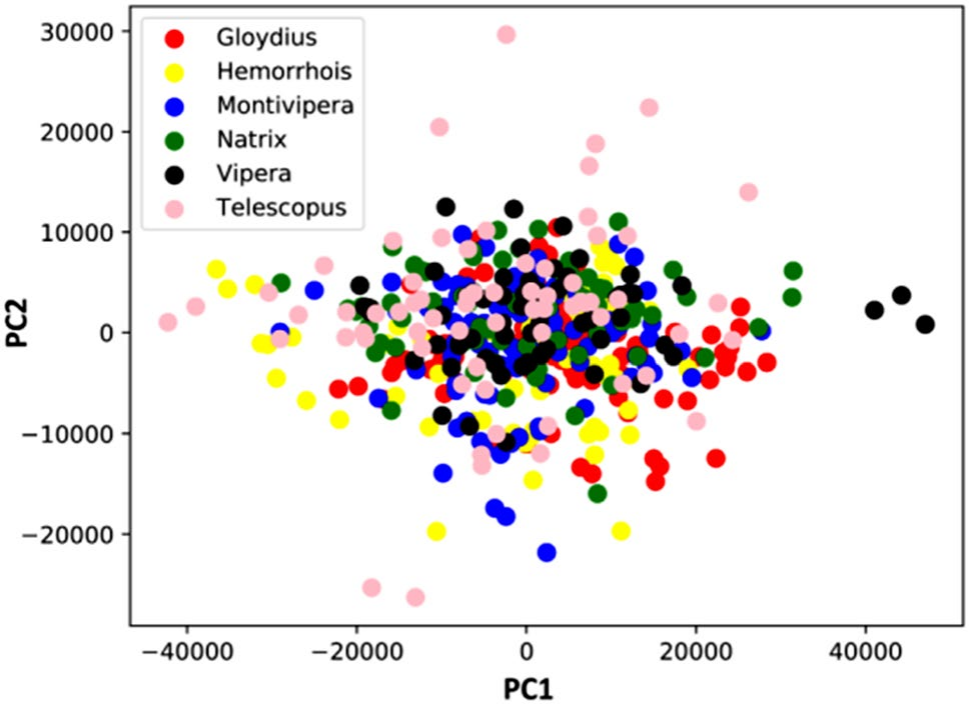
Scatterplot resulting from PCA over snake images.

PCA extracted 476 components. The first three components cumulatively explain 23.39, 25.83 and 26.78% of the total variance (Fig. 1).

### Linear discriminant analysis (LDA)

**Figure 2:**
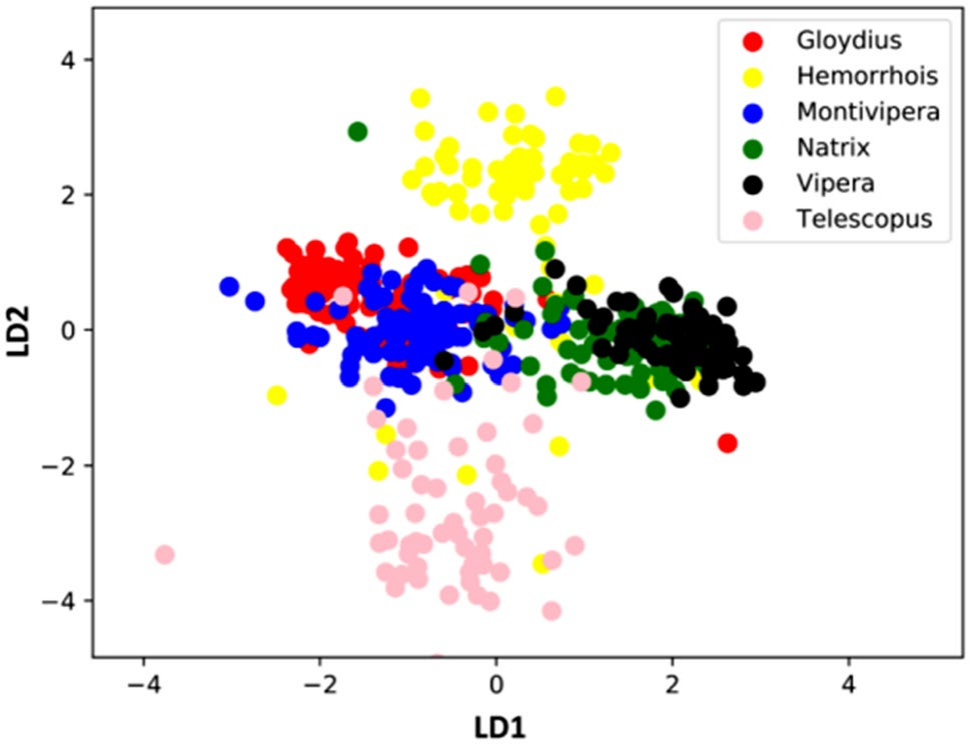
Scatterplot resulting from linear discriminant analysis (LDA) over the snake images.

LDA extracted five components; all of which cumulatively explain 28.22, 49.98, 70.49, 86.16 and 100% of total variance (Fig. 2).

### kNN classifier

**Figure 3:**
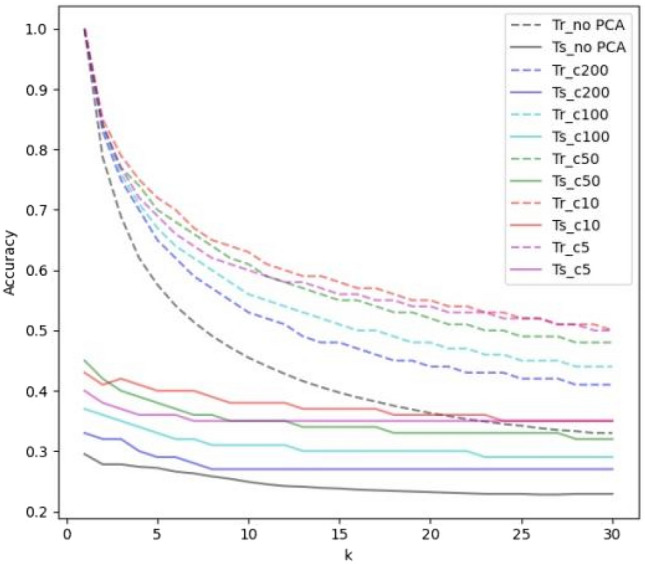
Results of snake image classification using *k*NN algorithm, as well as *k*NN in combination with a data dimension reduction approach (PCA), while *k* has been changing in a range from 1 to 30. The number of the components (c) is 5, 10, 50, 100 and 200.

**Figure 4:**
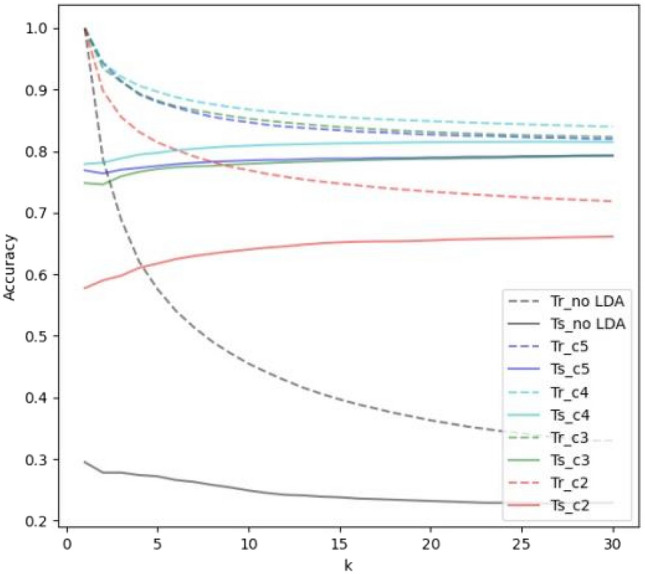
Result of snake image classification using *k*NN algorithm, as well as *k*NN in combination with a data dimension reduction approach (LDA), while k has been changing in a range from 1 to 30. The number of components (comp) was ranging from 2 to 5.

The accuracy of *k*NN algorithm used for the classification of snake images, merely and in combination with a data dimension reduction approach, is presented in Figs. 3 and 4; while *k* in these procedures has been changing in a range from 1 to 30.

### SVM

The result of snake image classification using SVM algorithm, both alone and in combination with a data dimension reduction approach, is presented in Table 1.

**Table 1:** Result of snake image classification using SVM algorithm.

Classifier (SVM)	SVM_LDA (Nr. components: 2, 3, 4, 5)		SVM_PCA (Nr. components: 2, 5, 10, 20, 50, 100, 200)		SVM	
	Tr	Ts	Tr	Ts	Tr	Ts
kernel = 'linear'	0.68, 0.77, 0.83, 0.83	0.66, 0.76, 0.81,0.81	–,–,–,–,–, 1, 1	–,–,–,–,–, 0.31, 0.30	1.00	0.40
kernel = 'poly', dr = 5	0.55, 0.63, 0.72, 0.72	0.52, 0.60, 0.68, 0.64	0.31, 0.45, 0.49, 0.52, 0.44, 0.55, 0.60	0.27, 0.34, 0.36, 0.33, 0.31, 0.29, 0.27	1.00	0.34
kernel = 'rbf'	0.71, 0.79, 0.87, 0.87	0.69, 0.76, 0.83, 0.84	0.36, 0.50, 0.57, 0.65, 0.73, 0.79, 0.83	0.31, 0.41, 0.42, 0.41, 0.45, 0.44, 0.43	0.93	0.43
kernel = 'sigmoid'	0.52, 0.63, 0.77, 0.79	0.50, 0.61, 0.76, 0.79	0.16, 0.20, 0.21, 0.23, 0.28, 0.34, 0.43	0.14, 0.18, 0.20, 0.21, 0.22, 0.23, 0.26	0.20	0.20

### Logistic regression

The result of snake image classification using logistic regression algorithm, both alone and in combination with a data dimension reduction approach, is presented in Table 2.

**Table 2:** Result of snake image classification using logistic regression algorithm.

	LR_PCA (Nr. components: 2, 5, 10, 20, 50, 100, 200)		LR_LDA (Nr. components: 2, 3, 4, 5)		LR	
	Tr	Ts	Tr	Ts	Tr	Ts
tol = 1e−2	0.25, 0.31, 0.36, 0.43, 0.57, 0.88, 1.0	0.23, 0.30, 0.30, 0.33, 0.32, 0.31, 0.35	–, –, 0.77, 0.84	–, –, 0.76, 0.83	1.0	0.35
tol = 1e−3	0.25, 0.28, 0.33, 0.41,0.56, 0.88, 1, 0	0.24, 0.27, 0.27, 0.30, 0.30, 0.30, 0.32	–, –, 0.77, 0.85	–, –, 0.78, 0.84	1.0	0.37
tol = 1e−4	0.25, 0.27, 0.33, 0.40, 0.56, 0.85, 1.0	0.23, 0.24, 0.26, 0.28, 0.31, 0.27, 0.34	–, –, 0.78, 0.82	–, –, 0.77, 0.81	1.0	0.36

### CNN

The VGG-16 model involves 134.260.544 parameters. The model was trained for 500 epochs. Besides, the MobileNetV2 involves 5.147.206 parameters and was trained for 150 epochs. Both the models were set up with SGD optimizer and a learning rate equal to 0.0001, as well as a momentum equal to 0.9 (Table 3).

**Table 3:** Confusion matrix showing the performance of MobileNetV2 and VGG-16 model for snake image classification of the test dataset. Total number of the samples has been presented bellow each column.

	MobileNetV2						VGG-16					
	G	H	M	N	V	T	G	H	M	N	V	T
*Gloydius*	22	0	1	2	0	0	20	2	1	1	0	1
*Hemorrhois*	1	16	0	0	0	1	1	16	0	0	0	1
*Montivipera*	0	0	24	1	0	0	0	1	21	1	0	2
*Natrix*	0	1	0	18	0	0	2	2	2	9	0	4
*Viper*	0	0	0	1	13	0	1	1	0	0	12	0
*Telescopus*	0	0	0	0	0	16	1	0	1	1	0	13
Total	25	18	25	19	14	16	25	18	25	19	14	16

**Figure 5:**
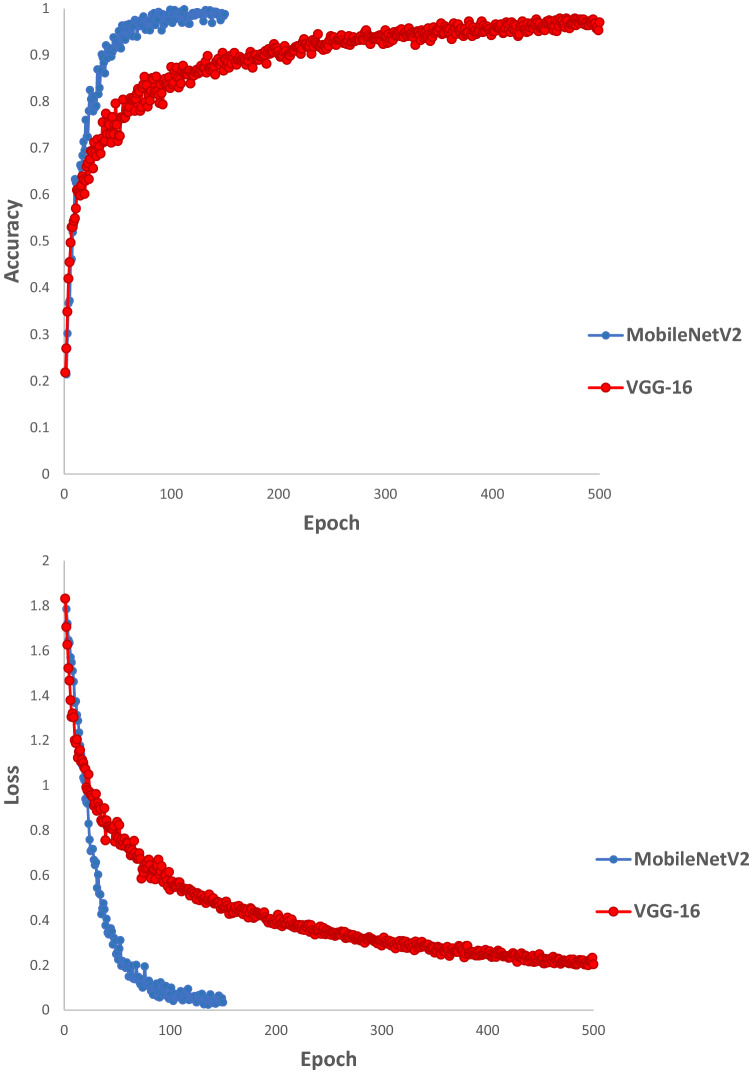
Accuracy and loss values of MobileNetV2 and VGG-16 model with an initial weight from ImageNet during the training process.

The models were run twice; once without an initial weight and another time with an initial weight from ImageNet. The models without the initial weight were not trained properly during the training process. In VGG-16, the train and test accuracy of the weighted model after one run reached to 96.82 and 77.78%; while in MobileNetV2 the train and test accuracy of the weighted model reached to above 90%. Hence a fivefold validation set were performed for MobileNetV2 and the accuracy obtained for the train and test of the model were 99.16 and 89.99%, 99.16 and 93.33%, 99.78 and 93.33%, 99.58 and 92.50%, and finally 100.0 and 91.67% (Fig. 5). MobileNetV2 model is a relatively robust model and induced noise in the input test images does not reduce the accuracy of the model drastically (Table 4). The Detailed result of snake image classification, using VGG-16 and MobileNetV2 algorithm, has been presented in a confusion matrix (Table 3); moreover an accuracy results table has been presented in Table 5.

**Table 4:** The F1-score and overall accuracy of MobileNetV2 model for modified test images datasets (augmented via width shift as well as vertical flip and zooming) showing the robustness of the model.

		
	Width shifted	Flipped and zoomed
	F1-score	F1-score
*Gloydius*	0.89	0.84
*Hemorrhois*	0.89	0.73
*Montivipera*	0.9	0.85
*Natrix*	0.87	0.75
*Vipera*	0.96	0.83
*Telescopus*	0.94	0.89
Accuracy	0.91	0.82

**Table 5:** Accuracy result of MobileNetV2 and VGG-16 model for snake image classification.

	MobileNetV2			VGG-16		
	Precision	Recall	F1-score	Precision	Recall	F1-score
*Gloydius*	0.96	0.88	0.92	0.8	0.8	0.8
*Hemorrhois*	0.89	0.89	0.89	0.73	0.89	0.8
*Montivipera*	0.96	0.92	0.94	0.84	0.84	0.84
*Natrix*	0.82	0.95	0.88	0.75	0.47	0.58
*Vipera*	1	0.93	0.96	1	0.86	0.92
*Telescopus*	0.94	1	0.97	0.62	0.81	0.7
Accuracy			0.93			0.78

**Figure 6:**
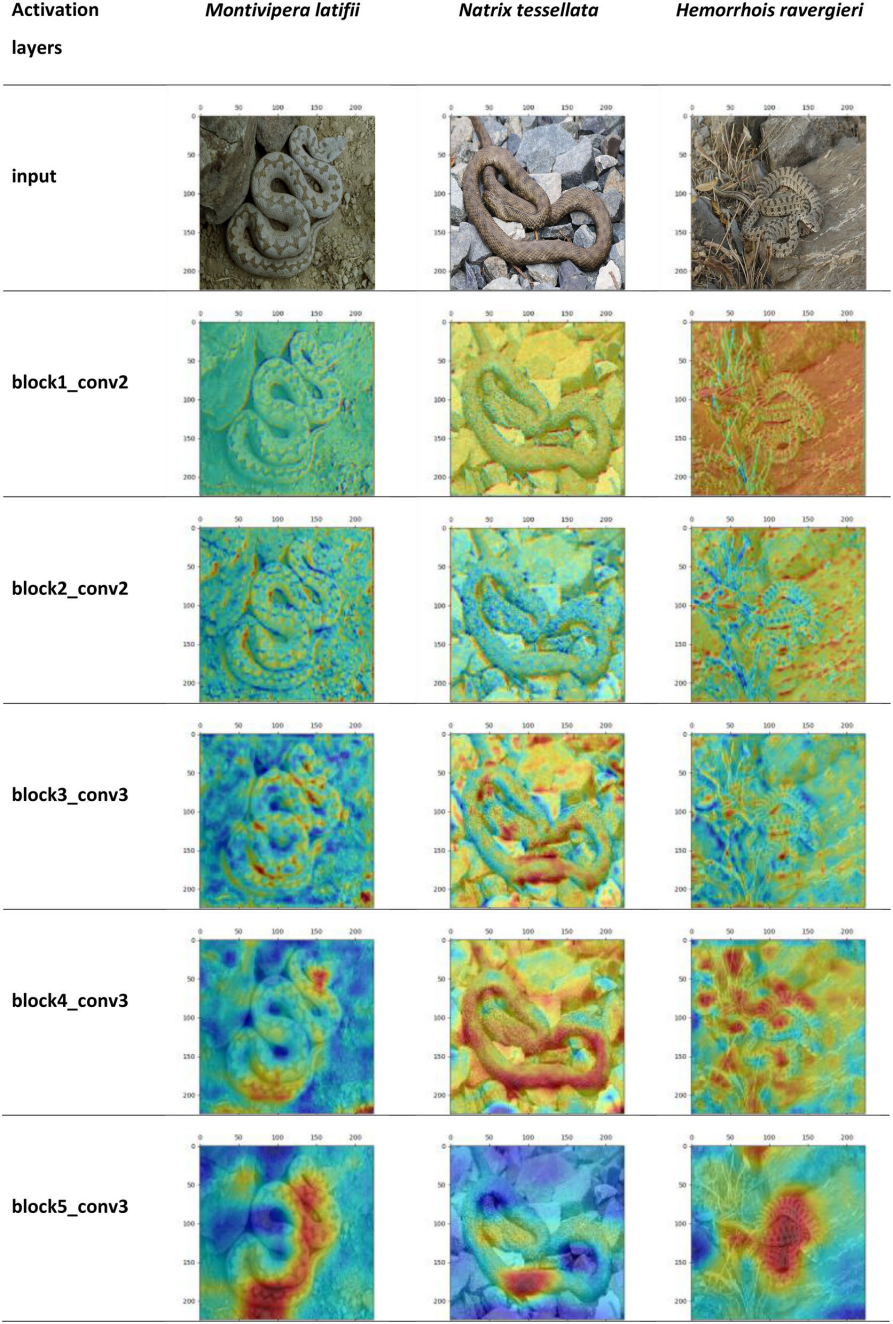
The heatmap visualization of discriminative regions within the hidden activation layers of VGG-16 model.

Visualizing the intermediate activation layers in VGG-16 (and similarly MobileNetV2) model revealed that although a snake and its environmental features are considered together via the model's filters in the first and second blocks of the activation layers toward the deeper layers, the model focuses on the dorsal pattern features of the snakes as the discriminant feature (Fig. 6).

## Discussion

In this study, for the first time, a series of state-of-the-art machine learning algorithms were compared in the classification of six snake species of Lar National Park. In holistic methods (*k*NN, SVM, LR), performance of the classifiers over the raw images’ data were not satisfying and the test accuracy did not exceed 50%. Application of the dimension reduction algorithms had different outputs; Application of PCA did not improve the accuracy of the model, but the use of LDA in extracting the important features significantly improved the performance of classifiers. A combination of LDA and SVM (kernel = 'rbf') reached a test accuracy of 84%.

Independent comparative studies of PCA and LDA on the FERET image datasets revealed that a particular combination of PCA or LDA with a classifier is not always the best combination for classification of each dataset, so choosing a dimension reduction approach depends on the dataset and the specific task^14^. Amir et al.^8^ used Color and Edge Directivity Descriptor (CEDD) as feature extractor. CEDD is a low-level feature that is extracted from images and might be used for indexing and retrieval^15^; Hence, in the classification of snake images of Perlis corpus dataset (including 349 images of 22 species of the snakes from Perlis Region in Malaysia), they used *k*NN (k = 1) and reached the accuracy of 89.22% (correct predictions).

James^9^ proposed a method that included manually cropping of 31 taxonomic features from snakes’ head and body images. Snake features are subsequently classified using the proposed method based on *k*NN algorithm. He used this method for classification of Elapid and Viperid snakes (two classes) and obtained the accuracy of 94.27%.

Compared to the holistic methods, the performance of neural network algorithms in image classification of snakes of Lar National Park was not the same. Although the performance of VGG-16 (Table 5) was not different than the holistic methods, but the image classification accuracy improved drastically using the MobileNetV2 (93.16%). the convolutional neural networks showed better performance than the holistic methods for image-based fish species’ classification^16^ and also plant leaf disease^17^. But opposite results are also reported, e.g*.* in auto identification of bird images^18^.

Patel et al.^10^ used a region-based convolutional neural network (R-CNN) for the detection and classification of nine snake species in Galápagos Islands and Ecuador; and using ResNet algorithm, they obtained an accuracy of around 75%, and using VGG-16, they obtained an accuracy of around 70% (Table 6).

So, in this paper we present MobileNetV2, as a powerful deep convolutional neural network algorithm for snake image classification, with an accuracy over 90%. Since MobileNetV2 is a light algorithm with few number of parameters, it could be used even on mobile devices. This possibility could be used in a mobile application that would be helpful e.g. in auto-identification of snake images by healthcare providers to help in snake bite management. Although the majority of the images used in this study come from SLR cameras, but to feed the classification model, the images were originally resized to 224*224 pixels that is far bellow the quality of images of modern smartphone camera. So, smartphone images could be properly used for training a MobileNetV2 model too.

**Figure 7:**
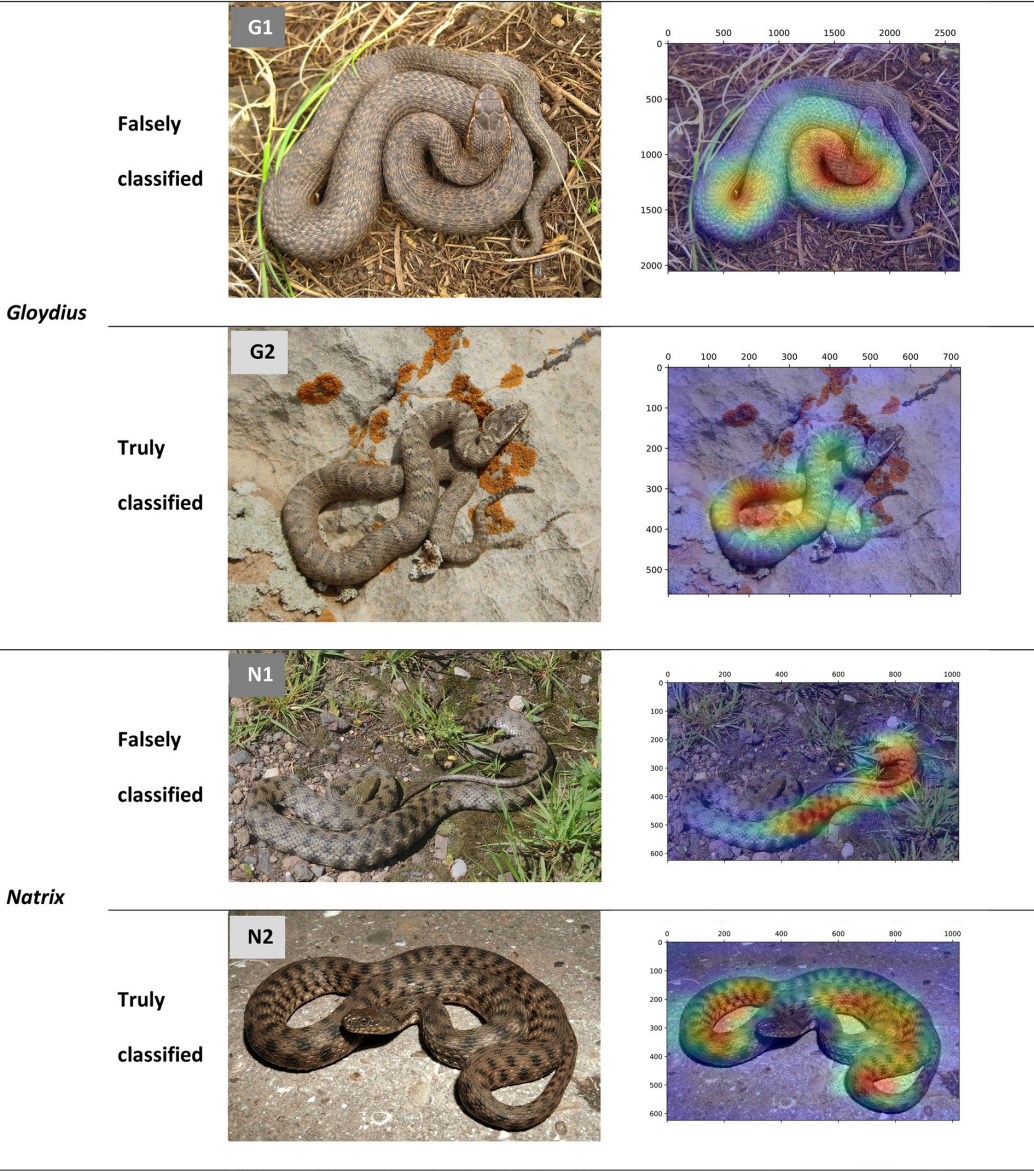
The heatmap visualization of discriminative regions within the last activation layers of VGG-16 model, in truly and falsely classified images of *Gloydius* and *Natrix* snakes.

**Figure 8:**
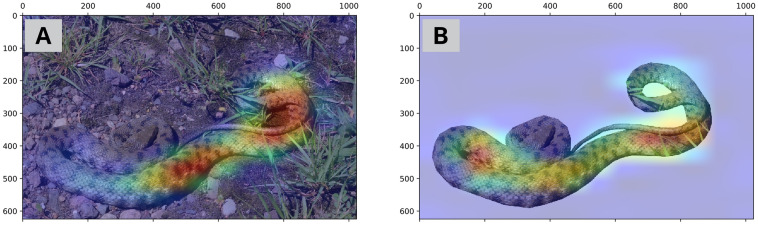
The heatmap visualization of the discriminative regions within the last activation layers of VGG-16 model, in *Natrix* (**A**: correspond to N1 in Fig. 7) snake, after removing the natural substrates (**B**).

Visualization of intermediate activation layers in VGG-16 (and similarly MobileNetV2) model reveals that the model mainly focuses on dorsal color pattern of snakes for a proper classification. Dorsal color pattern is a taxonomic key feature in the identification of snakes^1^. Looking at those snake images that are misidentified, and comparing them with the similar images that are truly classified (Fig. 7) reveals that in the misclassified images, the dorsal color pattern has not received a proper attention by the VGG-16 algorithm. This problem might have raised from the following cases:

- Dorsal pattern is not discriminative enough to identify the snake. For example, in a *Gloydius* snake (Fig. 7, G1), dorsal pattern is less pronounced than other specimens of the *Gloydius* (Fig. 7, G2), probably because the photographed snake was close to shedding and its dorsal pattern was somehow masked. Similar reason for misidentification was observe in classification of vector mosquitoes. Park et al.^19^ observed that if the lighting condition of the mosquito images are not good enough to clearly show the discriminating color features of the mosquitoes, the CNN model cannot identify them properly.
- Only a part of the snake’s dorsal pattern has received attention by the model. For example, in Fig. 7 (N1 and N2), the dorsal patterns are discriminative; whereas in N1 that only part of the pattern has received proper attention, the image is misidentified. This probably resulted from the cryptic effect of snake over its natural environment; hence, when the environment is removed from the image (Fig. 8, the overall color pattern of the snake receives more attention by the model. In classification of Chinese butterfly Xi et al.^20^ showed that image background removal enhanced model generalizability and provide a better results for test datasets.

Transfer learning is usually applied when a new dataset smaller than the original dataset is used to train the pre-trained model. ImageNet is an optimum dataset, but collecting enough number of images from the living organisms, especially not common ones, like many snake species, is not usually possible. Transfer learning greatly helps generate high accuracy models for the identification of living creatures. This technique was used successfully in generating models for the identification of e.g. vector mosquitoes^19^ and fish species^21,22^ too.

## Materials and methods

### Study area

The study area is Lar National Park, located in the northeastern Tehran Province, Iran. The park is a natural attraction, adjacent to Damavand Summit, where many visitors come for picnic, hiking, climbing, fishing etc.in springs and summer.s As well, nomad families and beekeepers reside in the area during warm seasons. Six snake species have been reported in Lar National Park, including three venomous, one semi-venomous, and two non-venomous snakes^1^ (Table 6)

**Table 6:** Diversity of snakes of Lar National Park (following^1^).

Nr	English name	Scientific name	Venom	Lethality	Human conflict
1	Alburzi viper	*Viperaeriwanensis*	Venomous	Low (LD50: 21.7)	Low
2	Caucasian pit viper	*Gloydiushalyscaucasicus*	Venomous	Medium (LD50: 13.6)	High
3	Dice snake	*Natrixtessellate*	Non-venomous	–-	High
4	European cat snake	*Telescopusfallax*	Semi-venomous	Very low	Medium
5	Latifi's viper	*Montiviperalatifii*	Venomous	High (LD50: 5.5)	Medium
6	Spotted whip snake	*Hemorrhoisravergieri*	Semi-venomous	–-	High

.

### Dataset

**Figure 9:**
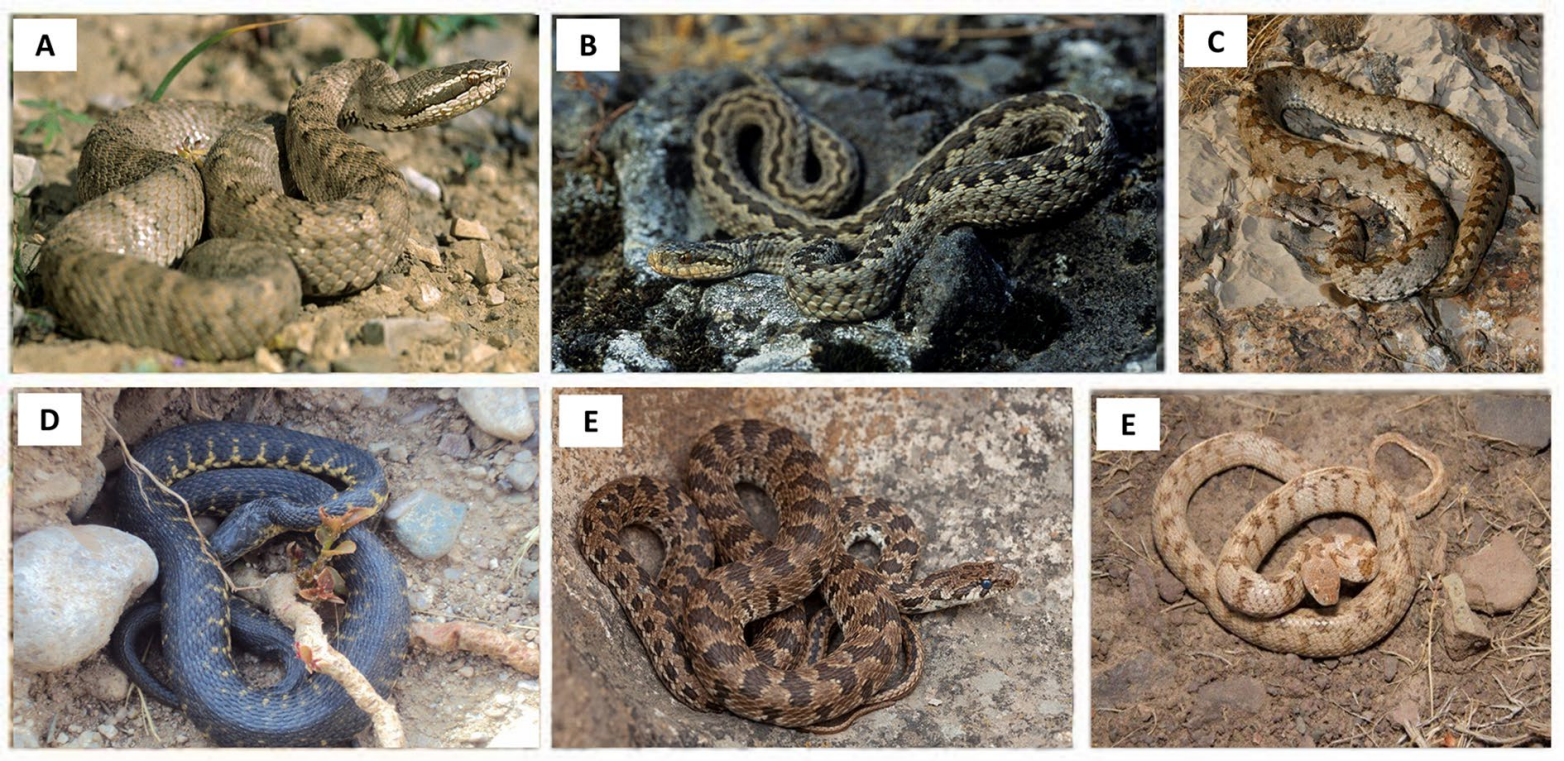
Samples of the images of the six snake species of Lar National Park. A: Caucasian pit viper, venomous; B: Alburzi viper, venomous; C: Latifi's viper, venomous; D: Dice snake, non-venomous; E: Spotted whip snake, non-venomous; F: European cat snake, semi-venomous.

Totally, 594 images of the six snake species of Lar National Park were collected, including 124 images of Caucasian pit viper, 80 of Alburzi viper, 124 of Latifi's viper, 95 of dice snake, 90 of spotted whip snake, and 81 of European cat snake (Fig. 9). The images were collected from personal archives (see the acknowledgement) and web databases, including https://www.calphotos.berkeley.edu and https://www.flickr.com. The images are of different sizes, with 24 bit RGB channels.

### Models

A series of state-of-the-art machine learning algorithms were used to compare their performed. This comparison was performed because in classification of biodiversity elements, depending on the subject, the performance of different classification algorithms may vary a lot and although for some taxa, deep learning algorithms show better performance^16,17^, for other taxa, shallow learning algorithms work better^18^. The methods are as follows:

#### Traditional or holistic methods

These methods represent images using the entire image region^23^. In this research, the holistic methods were used in combination with a dimension reduction approach. Projecting images onto a low-dimensional space is used to extract the important features and to discard the redundant details that are not needed for image classification^23^. Feature extraction could be defined as the act of mapping the image from image space to the feature space^4^. Among the popular approaches in this category, principle component analysis (PCA) and linear discriminant analysis (LDA) were used.

##### Principal component analysis

PCA is an unsupervised linear technique that uses an orthogonal transformation to project a set of variables into a lower dimension with maximum variance. In PCA a set of variables that are possibly correlated, convert into a set of values that are not correlated variables, called principal components^24^. To reduce each data *x*_*i*_* ∈ R*^*n*^ to y_*i*_* ∈ R*^*d*^ while d ≪ n, the PCA tries to find orthogonal matrix *U ∈ R*^*n*×*d*^ so that the reduced data *y*_*i*_ = *U*^*T*^*x*_*i*_ have the maximum variance. It has been shown that this projection matrix *U ∈ R*^*n*×*d*^ consists of *d* eigenvectors corresponding to the first *k* large eigenvalues of the following covariance matrix.

1 $$C= \frac{1}{N-1}\sum {({x}_{i}-\overline{x })({x}_{i}-\overline{x })}^{T}$$

where $\overline{x }$ and N are mean and the number of data, respectively^25^.

##### Linear discriminant analysis

LDA is a supervised feature extraction method that is usually used for the classification problems. LDA extracts low dimensional features which have the most sensitive discriminant ability from high dimensional feature space^26^. LDA for each data *x*_*i*_ tries to find an orthogonal projector *U* by minimizing the within-class distance and maximizing the between-class distance of the projected data *y*_*i*_ = *U*^*T*^*x*_*i*_. Mathematically, If we consider the number of classes equal to *K* and consider the number of elements within the class *k* represented as *N*_*k*_, then the index of maximizing the between-class separation and minimizing the within-class separation, leads to the maximizing the following objective function named Fisher discriminant analysis (FD) as:

2 $$\text{J}\left(\text{W}\right)=\frac{trace \,({U}^{T}{S}_{B}U)}{trace \,({U}^{T}{S}_{W}U)}$$

Here, *S*_*W*_ is the within-class distribution and *S*_*B*_ is the between-class distribution of the original data, and they are defined as:

3 $${S}_{B}={\sum }_{k=1}^{K}{N}_{k}{\left({m}_{k}- m\right)\left({m}_{k}- m\right)}^{T}$$

4 $${S}_{W}=\sum_{k=1}^{K}\sum_{{x}_{n}\in {C}_{k}}{\left({x}_{n}- {m}_{k}\right)\left({x}_{n}- {m}_{k}\right)}^{T}$$

where *m* and *m*_*k*_ are the mean of total data and the mean of the class *k.* LDA tries to find the matrix *U ∈ R*^*n*×*d*^ to maximize Eq. (3). in this regard, each data of *x ∈ R*^*n*^ is linearly transmitted to a *d* dimension space, as *y* = *U*^*T*^*x* . We can show that the result of maximizing Eq. (3), is *d* eigenvector, corresponding to the biggest eigenvalue of the following Generalized eigenvalue problem (Eq. 5)^27,28^.

5 $${S}_{B}u= \lambda {S}_{W}u$$

#### Traditional or holistic classifiers

Three types of traditional or holistic classifiers have been used in this study as follows. The training process in these classification algorithms only consist of storing the feature vectors and labels of the training images.

##### k-nearest neighbor

*k*NN is among the simplest machine learning algorithms that can classify the samples (data) based on the closest training examples in the feature space^29^.

The most common distance function for *k*NN, used in the current study, is Euclidean distance (Eq. 6):

6 $$d\left( {x,y} \right) = \sqrt {\mathop \sum \limits_{i = 1}^{n} \left( {x_{i} - y_{i} } \right)^{2} } = \parallel x - y\parallel$$

**Figure 10:**
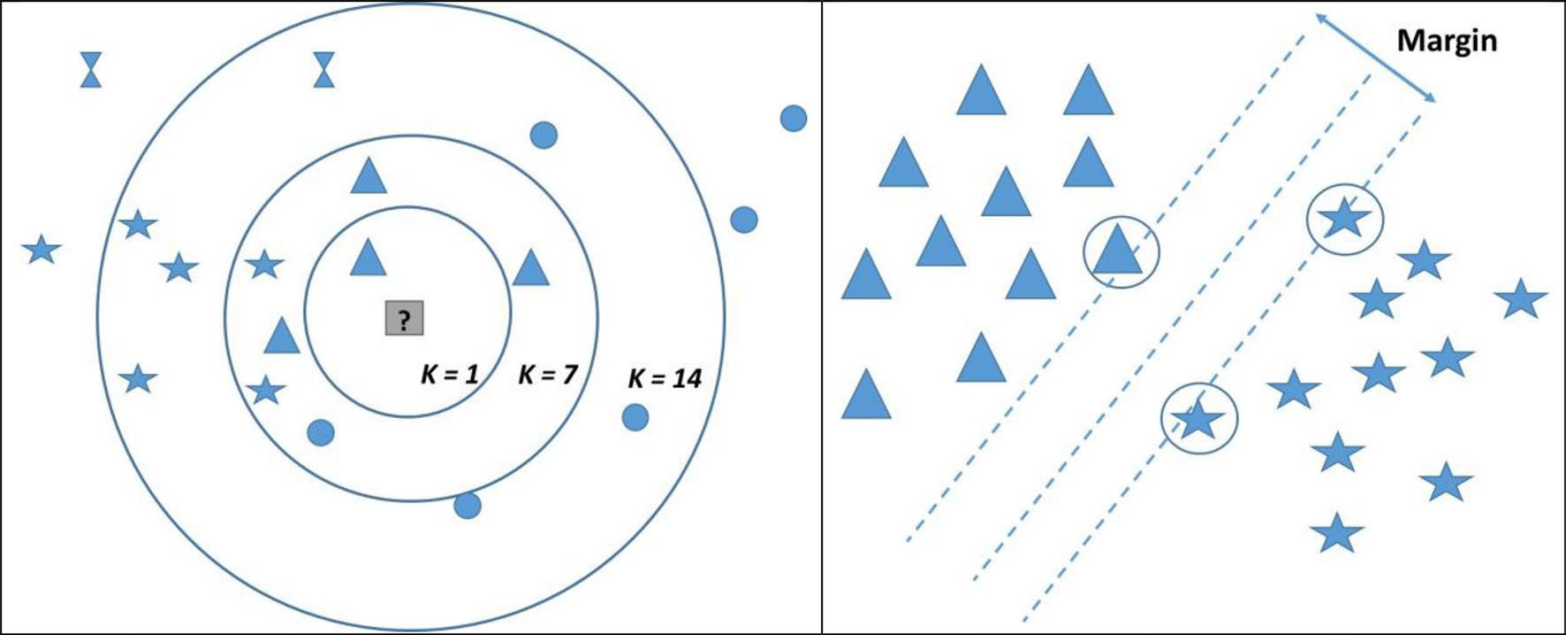
Left: A simplified, schematic drawing, showing a feature distance space and the classification process based on the nearest neighbors classifier. Given k = 1, the query image (question mark) is assigned to the label triangle. Right: A simplified, schematic drawing, showing the process of SVM classification. In multidimensional feature space, SVM finds the hyperplane that maximizes the margin between the classes (here two classes). Here, the support vectors are the circled labels.

During the classification process, using the *k*NN, the unlabeled query point is simply assigned to the label of its *k* nearest neighbors Fig. 10).

##### Support vector machines

SVM is a popular and powerful classification algorithm that can be used for image classification. Linear, Gaussian, Polynomial and Sigmoid kernel functions are used in developing SVM. The SVM tries to find two parallel hyperplanes as following

$${\omega }^{T}x+{\omega }_{0}=1$$

7 $${\omega }^{T}x+{\omega }_{0}=-1$$

With maximum distance from each other; each train data *x*_*i*_ satisfies the following equation

$$\forall {x}_{i} \in {C}_{1} \quad {\omega }^{T}{x}_{i}+ {\omega }_{0} \ge 1$$

8 $$\forall {x}_{i} \in {C}_{2}\quad {\omega }^{T}{x}_{i}+ {\omega }_{0} \le -1$$

Mathematically this problem leads to the following minimization problem

9 $$min\frac{1}{2} {\parallel \omega \parallel }^{2}$$

$${\mathrm{s}.\mathrm{t}. y}_{i}({\omega }^{T}{x}_{i}+{\omega }_{0})\ge 1$$

$$\mathrm{where }\quad {y}_{i}=\left\{\begin{array}{c}1\quad {x}_{i}\in {c}_{1}\\ -1 \quad {x}_{i}\in {c}_{2}\end{array}\right.$$

By writing the dual problem of this optimization, we have the following form:

10 $${Min\, L}_{D}=\sum_{i=1}^{n}{-\alpha }_{i}+ \frac{1}{2}\sum_{i=1}^{n}\sum_{j=1}^{n}{\alpha }_{i}{\alpha }_{j}{y}_{i}{y}_{j}{x}_{i}^{T}{x}_{j}$$

Subjected to

11 $$\sum_{i=1}^{n}{\alpha }_{i}{y}_{i}=0$$

By using kerners, this form gives a nonlinear version of SVM as follows:

12 $${Min \,L}_{D}=\sum_{i=1}^{n}{-\alpha }_{i}+ \frac{1}{2}\sum_{i=1}^{n}\sum_{j=1}^{n}{\alpha }_{i}{\alpha }_{j}{y}_{i}{y}_{j}k({x}_{i},{x}_{j})$$

Subjected to

13 $$\sum_{i=1}^{n}{\alpha }_{i}{y}_{i}=0, k\left({x}_{i},{x}_{j}\right):kernel$$

A more detailed discussion of the SVM has been presented in^30^.

##### Logistic regression

Logistic regression (hereafter LR) is a linear model that uses the cross entropy as a loss function, and is able to handle the outlier in the data.

#### Neural networks

Neural networks are described as a collection of connected units, called artificial neurons, organized in the layers. Neural networks can be divided into shallow (one hidden layer) and deep (more hidden layers) networks.

Feedforward neural networks is one of the most prevailing neural networks that is very popular for data processing^31^. But all the parameters in the Feedforward neural networks need to be tuned iteratively; besides, the learning speed of the networks is very slow, which limits its applications^32^. Huang et al.^33^ proposed a single hidden layer feedforward neural networks algorithm named extreme learning machine (ELM) that has faster learning speed. This algorithm is based on a new feedforward neural network training method, which assigns input weights and thresholds of the neuron weights randomly; and output weight needs to be calculated in the learning process^34,35^.

But for image recognition, convolutional neural networks (CNNs) are the most common type of deep learning method^36^.

**Figure 11:**
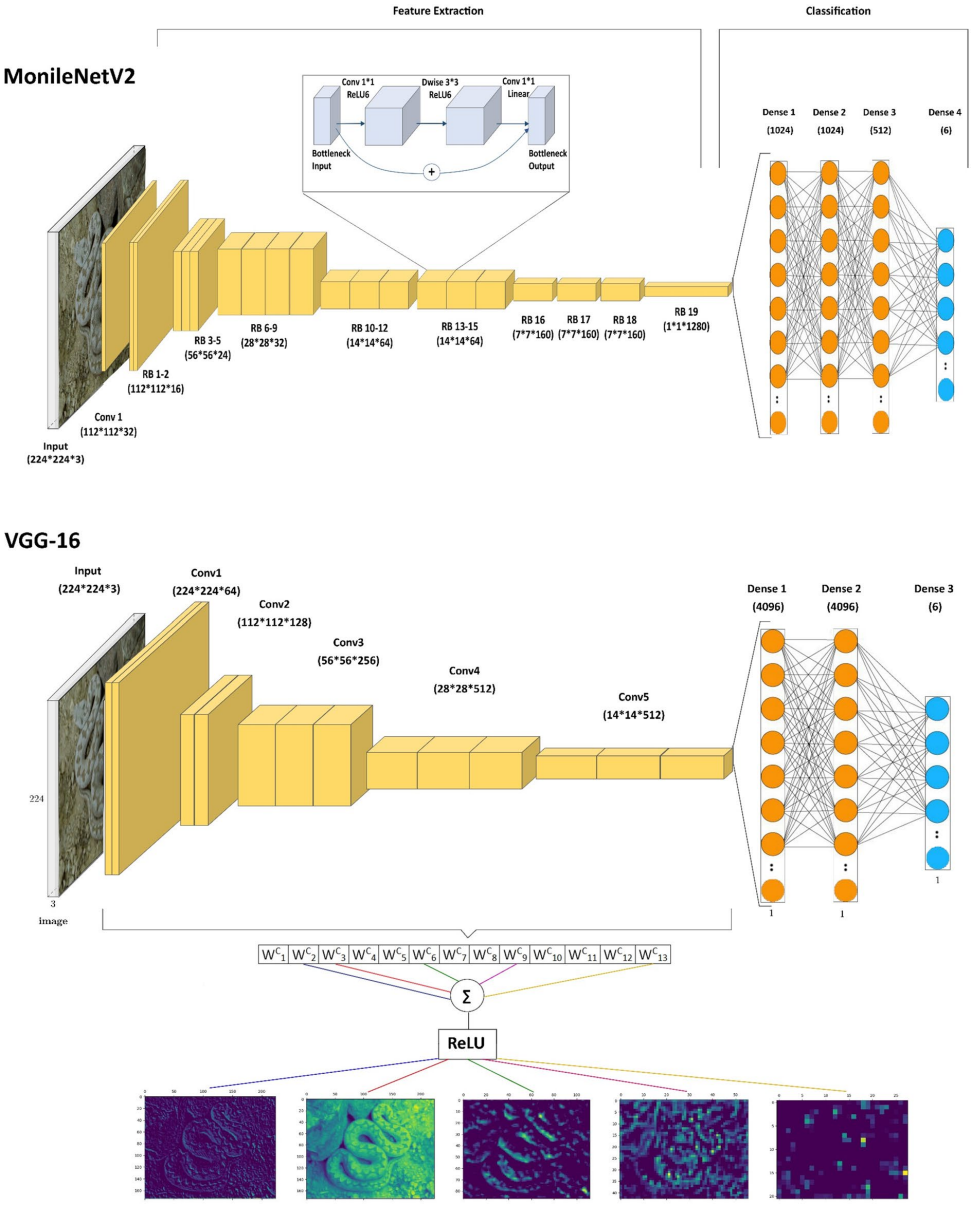
Architecture of the MobileNetV2 and the VGG-16 deep convolutional neural network^37^ (the image modified from^42^) as well as the process of visualization of hidden activation layers. *Cov* convolutional layer, *RB* residual bottleneck layer. Max pooling layers did not show to simplify the images.

Of deep neural networks, VGG-16^37^ and MobileNetV2^38^ algorithms are used in this paper. VGG-16 represents a memory-intensive deep learning model that has a large number of parameters, but its architecture is relatively simple and intuitive^37^ (Fig. 11). The architecture of MobileNetV2 is based on an inverted residual structure where the residual connections are between the bottleneck layers. The architecture of MobileNetV2 contains the initial fully convolution layer with 32 filters, followed by 19 residual bottleneck layers^38^. Despite the relative complexity in architecture, compared to other CNN models (including VGG-16), MobileNetV2 has considerably lower number of parameters that enable it to perform well even on mobile devices. We did not use more complex, residual based architectures like ResNet, as proposed in other literatures^10,39^, since ResNet has considerably high number of parameters and with our image dataset, the model was always subjected to overfitting.

#### Transfer learning

Transfer learning is an option for overcoming the limitations of input data for training a neural network model (to overcoming the limitations of input data for training a neural network model, transfer learning is an option). With transfer learning, those features extracted from the pre-trained networks are re-used for training a new neural network model. Else, transfer learning decreases the training time of a model. In this research, a VGG and a MobileNetV2 models are used for transfer learning. The models were first trained based on a dataset of ImageNet^40^, and then repurposed to learn features (or transfer them) on our dataset. In this way, the model obtained an initial weight from ImageNet. ImageNet is a dataset of over 15 million labeled high-resolution images belonging to roughly 22,000 categories^41^.

#### Discriminant features visualization

Visualizing each intermediate activation layer consists of displaying the feature maps that are produced by the convolution and pooling layers in the network. For this purpose, some recently developed visualization methods^13^ are used to locate the discriminative regions in the image output of the activation layers of VGG model (Fig. 11). These visualizations only were generated for VGG-16 since the architecture of this model is simpler and more understandable than MobileNetV2. Visualization was performed using the "keras" package^40^.

## Experiments

### Image preparation

All input images were resized to 224*224*3. Subsequently, each image was converted to a vector with a length of 150,528; afterward, the vectors were converted to a matrix with 594 rows and 150,528 columns. The input data (images) were partitioned to 80% for the training, and 20% for the test. Subsequently, for the holistic methods, the classification was performed with a tenfold validation set; each fold with different images in train and test, compared to other experiments, to prevent the overlapping of testing and training images in each experiment.

For the neural network methods, we used a series of data augmentation techniques for the train images; Hence, only the train images were randomly rotated in a range of 0 to 45 degrees and flipped both horizontally and vertically. To check the robustness of the neural network model, the test images were modified using a series of augmentation techniques (not used for the training images) and then using the augmented test images, the performance of the model were evaluated again.

### Models

The models were generated in Python (version, 3.8) using the "Scikit-learn" package^43^ and the "keras" package^40^ with TensorFlow^44^ as the backend. The analyses were performed on Google Colab.

### Performance metrics

The performance of the classification algorithms were evaluated using three metrics including the accuracy, precision and recall. The accuracy is simply defined as the fraction of correct predictions of the model to total number of the predictions. Accuracy can also be calculated in terms of positives and negatives as follows (Eq. 14):

14 $$Accuracy= \frac{TP+TN}{TP+TN+FP+FN}$$

where TP is true positives, TN is true negatives, FP is false positives, and FN is false negatives.

The precision (also called positive predictive value) is the fraction of test images classified as a class *A* that are truly assigned to the class *A* (Eq. 15); whereas recall (also known as sensitivity) is the fraction of test images from a class *A* that are correctly identified to be assign to the class *A* (Eq. 16).

15 $$Precision= \frac{TP}{TP+FP}$$

16 $$Recall= \frac{TP}{TP+FN}$$

The average of the precision and recall could be interpreted as F1 score, having its best value at 1 and worst value at 0 (Eq. 17).

17 $$F score= 2(\frac{Precision . Recall}{Precision+Recall})$$

To simplify the comparisons for the holistic algorithms, the performance of the models were presented solely based on the accuracy; but for a neural network algorithm the performance of the model was evaluated using the three metrics, the accuracy, precision and recall.
